# Novel Splicing of Immune System Thyroid Stimulating Hormone β-Subunit—Genetic Regulation and Biological Importance

**DOI:** 10.3389/fendo.2019.00044

**Published:** 2019-02-05

**Authors:** John R. Klein

**Affiliations:** Department of Diagnostic and Biomedical Sciences, School of Dentistry, The University of Texas Health Science Center at Houston, Houston, TX, United States

**Keywords:** alternative splicing, hormone, immune-endocrine, leukocyte, thyroid

## Abstract

Thyroid stimulating hormone (TSH), a glycoprotein hormone produced by the anterior pituitary, controls the production of thyroxine (T_4_) and triiodothyronine (T_3_) in the thyroid. TSH is also known to be produced by the cells of the immune system; however, the physiological importance of that to the organism is unclear. We identified an alternatively-spliced form of TSHβ that is present in both humans and mice. The TSHβ splice variant (TSHβv), although produced at low levels by the pituitary, is the primary form made by hematopoietic cells in the bone marrow, and by peripheral leukocytes. Recent studies have linked TSHβv functionally to a number of health-related conditions, including enhanced host responses to infection and protection against osteoporosis. However, TSHβv also has been associated with autoimmune thyroiditis in humans. Yet to be identified is the process by which the TSHβv isoform is produced. Here, a set of genetic steps is laid out through which human TSHβv is generated using splicing events that result in a novel transcript in which exon 2 is deleted, exon 3 is retained, and the 3′ end of intron 2 codes for a signal peptide of the TSHβv polypeptide.

## Introduction

### Structure and Function of TSH

TSH consists of an α and a β subunit. The β subunit determines hormone specificity. Binding of TSH to the TSH receptor (TSHR) in the thyroid induces the release of thyroxine (T_4_) and a limited amount of triiodothyronine (T_3_). In tissue cells, most T_4_ is converted by deiodination to T_3_, the more biologically-active form of thyroid hormone. Regulation of TSH release from the pituitary is governed by levels of circulating TSH, T_4_, and T_3_. Considerable homology exists at both the gene and protein levels between human and mouse TSHβ (1, 2). Within the *hypothalamus-pituitary-thyroid* (HPT) axis, TSH regulates a broad spectrum of cellular and organismic activities, including cell physiology, development, and cellular and organismic metabolism. The TSHβ polypeptide is coded for by exons 4 and 5 of the mouse TSHβ gene, and by exons 2 and 3 of the human TSHβ gene.

### Immune System TSH

The pioneering work of Blalock and colleagues was the first to demonstrate that TSH is made by the immune system. The authors reported that leukocytes cultured with known forms of immune stimuli, such as *Staphylococcus* enterotoxin A or TRH, secreted immunoreactive TSH (3–5). Other studies demonstrated that splenic dendritic cells from mice produced TSH endogenously, as well as following stimulation with *Staphylococcus* enterotoxin B (6, 7). Bone marrow (BM) hematopoietic cells, in particular CD11b^+^ monocyte/macrophage precursors and to a lesser extent granulocyte and lymphocyte precursors, were shown to be a source of TSH (8). Additionally, intraepithelial lymphocytes and lamina propria lymphocytes in the intestinal mucosa of mice produced TSH locally following experimental virus infection (9–11). In studies aimed at understanding the integration of TSH immune-endocrine interactions, we observed that BM-derived CD11b^+^ cells traffic to the thyroid where they are deposited around thyroid follicles (7), and produce TSHβ intrathyroidally (6). Those studies collectively provided evidence that immune system-derived TSH may co-regulate the synthesis and release of thyroid hormones under normal homeostatic conditions (7).

There is longstanding evidence linking TSH to immune system function. TSH has been shown to directly influence the cellular responses of leukocytes in both positive and negative ways. This includes enhancement of antibody responses to T cell-dependent antigens (12), and iodine uptake inhibition (13, 14). T_3_ stimulates thymocyte proliferation, which may be the consequence of TSH-mediated increase in thyroid hormones (15). In that light, recent studies have documented a protective effect of TSH on thymocyte apoptosis (16). These and other directional interactions of HPT hormones on immunity have been detailed in several review articles (17, 18).

## The TSHβ Splice Variant (TSHβV)

**Figure 1:**
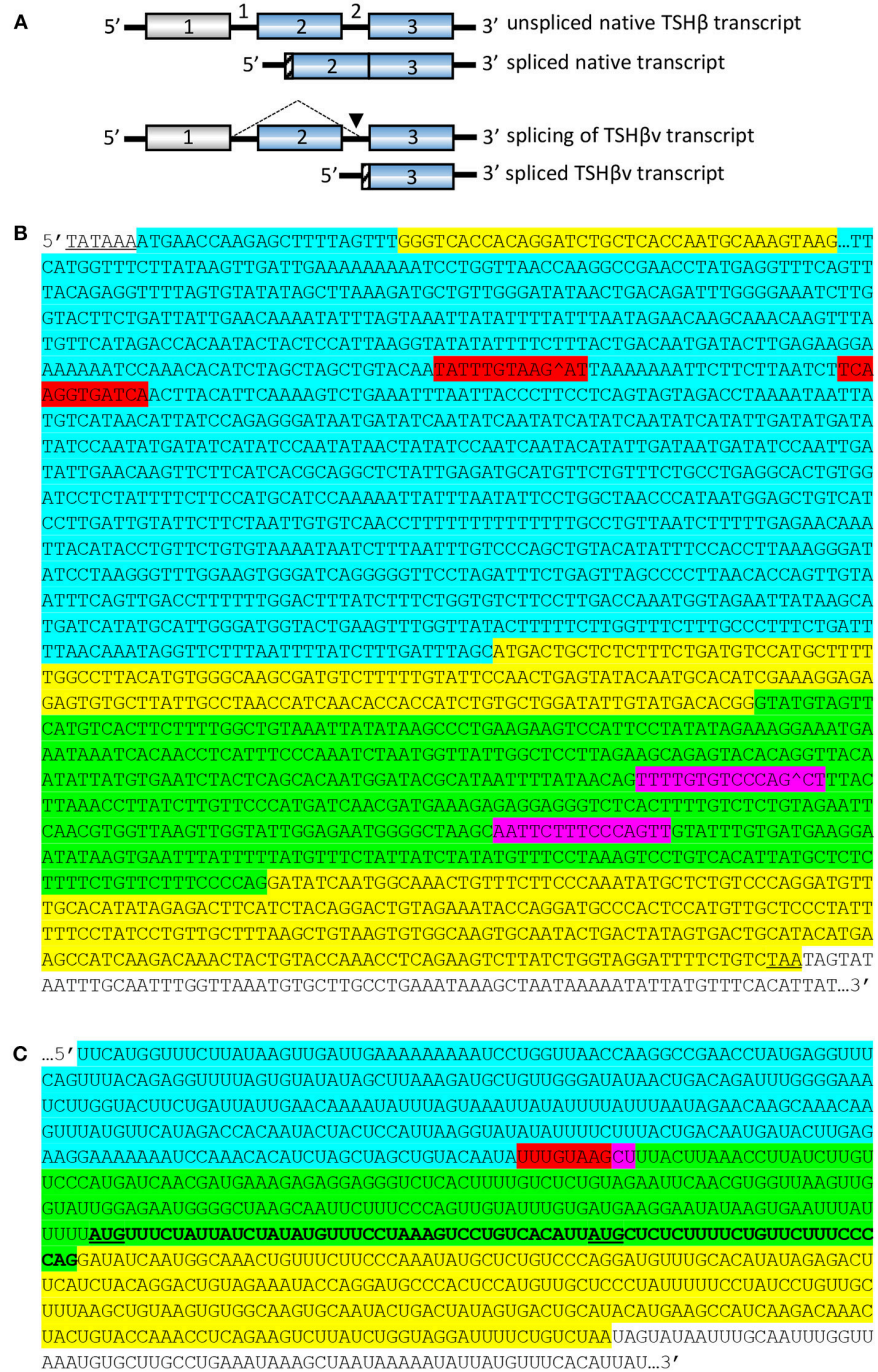
Structural organization and splicing patterns of the native and splice variant forms of human tshβ transcripts. **(A)** The TSHβv transcript is produced by removal of the region coded for by exon 2, and splicing into the 3′end of intron 2 (arrow), yielding a truncated TSHβ transcript coded for by exon 3. Gray boxes are transcript regions from non-coding exons. Blue boxes are transcript regions from coding exons. Hatched regions code for signal peptides, which in native TSHβ is at the beginning of exon 2, and in TSHβv is at the 3′ end of intron 2. **(B)** Unspliced TSHβ. Gray underlined nucleotides, TATAAA hexamer box of transcriptional start site. Upper yellow nucleotides, exon 1. Blue nucleotides, portion of intron 1. Middle yellow nucleotides, exon 2. Green nucleotides, intron 2. Bottom yellow nucleotides, exon 3. Red nucleotides, potential splice donor sites. Purple nucleotides, potential splice acceptor sites. Underlined TAA, stop codon at end of exon 3. White nucleotides, untranslated portion of exon 3. Potential donor sites in intron 1: TATTTGTAAGAT, TCAAGGTGATCA; both with donor site scores ≥83.4. Potential acceptor sites in intron 2: TTTTGTGTCCCAGCT, AATTCTTTCCCAGTT; both with acceptor site scores ≥88.0. **(C)** Spliced human TSHβ resulting in TSHβv transcript. Blue, intron 1-coded nucleotides. Green, intron 2-coded nucleotides. Yellow, exon 3-coded nucleotides. Combined red/purple, splice site of intron 1 with intron 2 resulting in deletion of exon 2-coded nucleotides but leaving exon 3-coded nucleotides. Bolded 69 nucleotide sequence in intron 2 codes for a putative 23 amino acid signal peptide of the TSHβv protein molecule with translation being initiated at the AUG codon (underlined). A second smaller signal peptide could be coded for beginning at the AUG (underlined) that is located 27 nucleotides upstream from the beginning of exon 3. White nucleotides, untranslated portion of exon 3.

Studies in our laboratory revealed that at both the transcript and protein levels, TSHβ produced in the thyroid as well as by leukocytes of mice and humans was significantly smaller in size than pituitary-derived TSHβ (19, 20), though it retained functional activity as determined by cAMP cell signaling (19). Further analysis of leukocyte-derived TSHβ identified a novel TSHβ splice variant (TSHβv) transcript coded for by exon 5 and the 3′ end of intron 4 in mice (19), and exon 3 and the 3′ end of intron 2 in humans (20, 21). Those splicing patterns for human native TSHβ and TSHβv are shown in Figure 1A.

Experiments using mouse BM cells confirmed that TSHβv is the principal form of TSHβ made by hematopoietic cells, particularly by CD11B^+^ myeloid cells (19, 21, 22). Similar findings were obtained using mouse splenic leukocytes (22). Zymosan, lipopolysaccharide, and CD14 and TLR2 triggering induced the release of TSHβv from the mouse AM macrophage cell line (22). Similarly, the THP-1 human macrophage cell line expressed the TSHβv gene, but not the native TSHβ gene (23). Interestingly, the TSHα gene was expressed at low levels in mouse BM cells (19) and human THP-1 cells (23), suggesting that TSHβv may operate either as a monomeric molecule or as a dimer. Further evidence of the latter is supported by studies demonstrating that TSHβv is dimerized with TSHα in the human circulation (24), a finding that is consistent with the presence of an eighteen amino acid “seatbelt” region coded for by human exon 3 that is used for attachment of TSHβ to TSHα (2). Thus, the TSHβv protein may under certain circumstances be coupled to TSHα.

Following polarization into M1 and M2 macrophages, TSHβv was primarily produced by M2 macrophages in mice (21), and by both M1 and M2 cells generated from the human THP-1 cell line (23). *In vivo* treatment of mice with T_4_ for 21 days resulted in differential effects on TSHβv expression in the BM and the pituitary, such that it was significantly increased in BM cells and suppressed in pituitary tissue (23). T_3_-treated F4/80^+^ RAW 264.7 mouse macrophages had dose-dependent increases in TSHβv expression (23).

### A Putative Basis of TSHβv Splicing

The findings described above demonstrate that the TSHβv isoform is selectively expressed in hematopoietic cells. An as yet unresolved question pertains to the process by which the isoform is generated. Given that the immune system makes TSHβv and little if any native TSHβ, there must be well-regulated process for doing this. Understanding the underlying mechanism of that will likely provide valuable insights into how TSHβv is functionally linked to the homeostatic balance of thyroid hormone synthesis, to disease, and/or to the control of host metabolic activity.

Alternative splicing is a complex process by which cells generate variable protein constructs from a common gene source (25, 26). Numerous mechanisms have been described through which alternative isoforms can be made. These include exon skipping, alternative use of 3′ or 5′ splice sites, and intron retention (27, 28). In hormone biology, the presence of receptor isoforms produced by alternative splicing is common, as is seen in secretin and gastrin receptors, for example (29). However, alternatively-spliced isoforms may produce receptors associated with neoplastic cell growth, as occurs in HER-2/neu (30).

In classical splicing situations involving intron removal, a spliceosome directs the splicing process by recognizing splicing signals in the transcript and catalyzing intron removal and exon joining (31). However, the process of alternative splicing for TSHβv differs notably from traditional approaches, including those in which exon skipping occurs, because a portion of the 3′ end of the intron preceding the last coding exon (exon 3 in humans and exon 5 in mice) is incorporated into the new isoform. Although utilization of introns in alternatively-spliced genes is known to occur (32), the majority of those retain the entire intron, as is the case with the tissue factor gene in which a new exon is generated from an intronal sequence. (33). Proteins in malignant tissues also have been shown to utilize intron inclusion (34). The reasons why this occurs are unclear, although they may be tied to mechanisms of dysregulated gene expression that leads to neoplasia (35). While there is no evidence of this for immune system TSHβv, it is possible that under some circumstances TSHβv may be expressed autonomously in the absence of known stimulatory signals (21).

**Figure 2:**
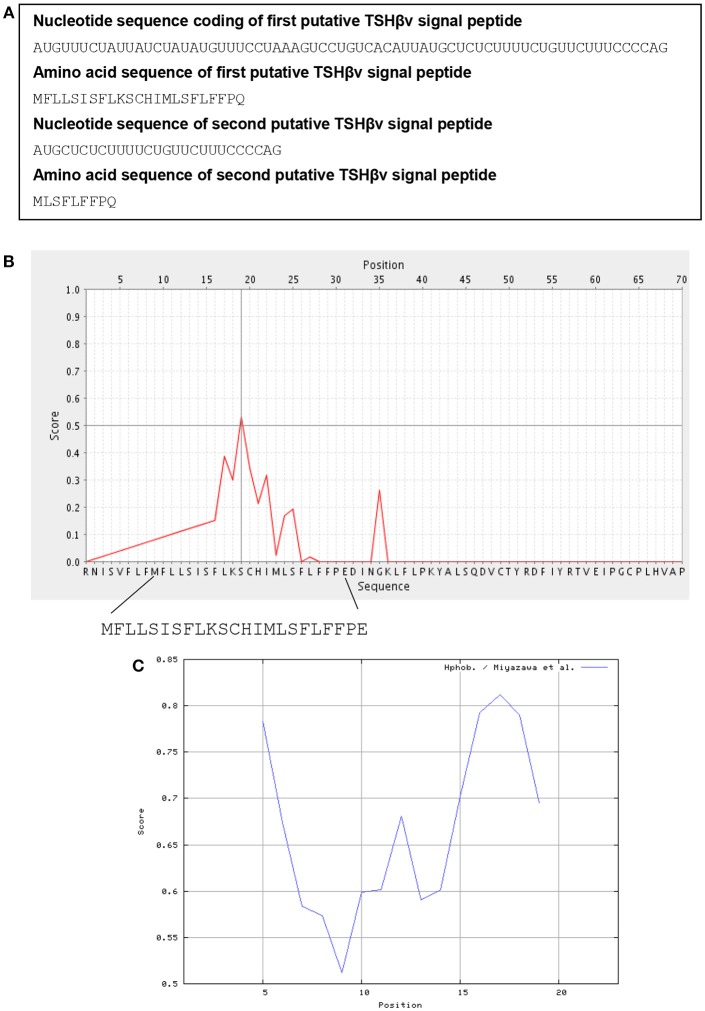
Signal peptide analysis of TSHβv in intron 2. **(A)** The nucleotide and amino acid sequences at the 3′ end of intron 2 (see Figure 1C, bold sequences) that code for a putative 23 and 9 amino acid signal peptides of TSHβv. **(B)** Biophysical properties of the putative 23 amino acid TSHβv signal peptide as determined using a PrediSi signal prediction program, the 23 amino acid peptide with the serine reside at position 11 likely representing the cleavage site. **(C)** The 23 amino acid signal peptide has hydrophobicity scores between 0.5 and 0.85 in a range from 0.0 (lowest hydrophobicity) to 1.0 (highest hydrophobicity).

The structure of the human unspliced TSHβ gene is shown in Figure 1B. Initiation of transcription is believed to occur at the TATAAA hexamer 22 nucleotides upstream from exon 1 (36). To gain insights into how TSHβv might be generated, the spliceport on-line prediction tool http://spliceport.cbcb.umd.edu/SplicingAnalyser.html was applied to the human unspliced TSHβ gene (36), from which a number of possible splice sites were identified. These consisted of donor sites in intron 1 and acceptor sites in intron 2 (Figure 1B). All four of those have predictive splicing scores ≥80.0. Note that splicing in using those would result in the complete deletion of exon 2, the portion of the TSHβ transcript that codes for the native TSHβ but not the TSHβv polypeptide. The final rearranged TSHβv transcript after splicing is shown in Figure 1C. This results in the retention of a piece of the 3′ end of intron 2 that may code for two possible signal peptides, one beginning with an AUG translational start site located at position −69 from the 3′ end of intron 2, the second located −27 nucleotides from the 3′ end of intron 2 (Figure 1C, underlined AUGs). These would code for 23 and 9 amino acid peptide sequences, respectively, as shown in Figure 2A.

### Signal Peptide Analysis of TSHβv

Using on-line signal peptide prediction tools http://www.predisi.de/, the 23 amino acid peptide shown in Figure 2A, prior to and contiguous with exon 3, bears physiochemical properties that are typical of signal peptides as shown by the large number of hydrophobic and polar non-charged residues (Figures 2B,C). Peptide sequences such as those are commonly used to feed mature proteins through the membrane translocon for extracellular secretion. Although the nine amino acid signal peptide would be substantially shorter, it too retains most of the elements needed for a signal peptide. Which of those two is preferentially used may eventually be predicted from sequence analyses studies of TSHβv. While neither of the translational start sites contain classic Kozak features, a number of new mechanisms for initiating translation has been identified in eukaryotic cells (37), thus making it conceivable that either one of the two could serve as signal peptides for human TSHβv.

### Transcriptional Regulation of TSHβv

The predictions presented here provide a potential process whereby the human TSHβv isoform may be generated in cells of the immune system. Still to be learned is the process that regulates when TSHβv is made, given the selective use of that form of TSHβ by leukocytes. Although transcription could be initiated in intron 2 at the standard transcriptional start site used for the native TSHβ gene, it also could begin with the TATAA box in intron 3, (see Figure 1B). Early studies into TSHβ gene regulation identified Pit-1 and GATA-2 as potential transcription factors acting on the TSHβ promoter in both rat and human TSHβ (38–42). However, it has since been learned that Pit-1 is probably minimally involved, and that GATA-2 may be the primary activator of TRH-driven transcription of the TSHβ gene (43–46). Other studies have shown that thyroid hormone negatively regulates TSHβ by interfering with the binding of transcription factors near the proximal promoter region (47, 48). In the case of immune system TSHβv, no transcription factors have, as yet, been linked to gene activation.

### Biological Significance of TSHβv

Although it is now clear that TSHβv is made by cells of the immune system, many unanswered question remain regarding the functional role of the TSHβv isoform (49), and why the production of it is primarily relegated to the cells of the immune system rather than as an isoform made in the pituitary through the conventional HPT axis. In that vein, the immune system is by nature involved in sensing and responding to a wide range of external stimuli, in particular to a variety of threats to the safety and well-being of the host. An example of this is the situation of acute or chronic immune stress due to infection. Although some TSHβv-producing spleen cells routinely migrate to the thyroid in healthy animals (7), our studies demonstrated a marked increase in TSHβv gene expression in the thyroid of mice following virus infection with reovirus (19) or bacterial infection with *Listeria monocytogenes* (22). In the case of the latter, trafficking of splenic CD14^+^, Ly6C^+^, Ly6G^+^ cells to the thyroid was accompanied by robust intrathyroidal TSHβv synthesis (22). Importantly, splenic leukocytes from *L. monocytogenes*-infected mice homed to the thyroid when injected into non-infected mice, thus pointing to an active and integrated immune-endocrine host response during infection (22). We have speculated that this constitutes an immune system-driven host response used to manipulate, specifically to curtail, metabolic activity at a time when energy conservation is crucial (50).

Additional work is needed to understand the significance of TSHβv in terms of thyroid hormone synthesis and metabolic regulation, as well as to elucidate its action within the thyroid. This could occur if the TSHβv polypeptide, either as a monomer or as a TSHα/TSHβv dimer, interfered with the binding of native TSHβ to the TSHR. It is known, for example, that naturally-occurring minor biophysical changes in the human TSHβ subunit can have dramatic effects on the ability of TSH to bind to the TSHR (51–53). Given that the human TSHβv polypeptide retains the seat-belt region coded for by exon 3 that is used to couple to TSHα, a TSHα/TSHβv complex would almost certainly exhibit altered physiochemical properties that could substantially affect the binding of the conventional TSHα/TSHβ complex to the TSHR. Additional studies will be needed to understand the effects of competitive binding between TSHβv vs. native TSHβ in driving the release of thyroid hormone. Moreover, because thyroid hormones directly influence peripheral leukocyte function, as has been shown for neutrophils (54, 55), monocytes and macrophages (56), and dendritic cells (57), a loop may exist between TSHβv produced by the immune system, and the regulatory effects of TSHβv-mediated thyroid hormone release on the immune system itself.

Immune system TSHβv has been tied to the host response in other physiological scenarios. Various studies have implicated low TSH levels, such as occurs in hyperthyroidism, with an increased risk of osteoporosis (58, 59). This is reinforced by studies demonstrating a reduction in bone loss concomitant with TSH binding to TSHRs on bone osteoblasts (60). Perhaps most striking, however, was the finding that the TSHβv made by bone marrow cells has an osteoprotective effect by acting directly on bone TSHRs. Thus, TSHβv may play a key role in bone health (21, 61–63).

In addition to the use of immune system TSHβv in response to infection and for bone remodeling, a number of human conditions tied to thyroid dysregulation, many of which are associated with inflammation or autoimmunity, have yet to be fully understood. These include Graves' disease and Hashimoto's thyroiditis (64–66), Graves' Ophthalmopathy (67, 68), Pendred's Syndrome (69), Lyme disease (70), inflammatory bowel disease (71), rheumatoid arthritis (72), systemic lupus erythematosus (73, 74), psoriasis (75), asthma (76), and sepsis (77, 78). Of particular interest, patients with Hashimoto's thyroiditis (HT) were found to have elevated transcript levels of TSHβv in PB compared to normal controls (24). Treatment of HT patients with prednisone reduced TSHβv transcript levels in patients having disease of < 9 months compared to patients with a disease >18 months (24). Also in that study, dexamethasone treatment suppressed in a dose-dependent fashion the expression of TSHβv in PBLs of HT patients (24). A second study implicated plasma cell-derived TSHβv in the pathogenesis of HT (79).

## Summary

A model is presented in which the TSHβv transcript is produced in cells of the immune system using a mechanism of alternative splicing that results in deletion of exon 2 and retention of exon 3 coded portions of the transcript. A unique feature of this splicing process is that the transcript codes for a signal peptide derived from the 3′ end of intron 2 prior to exon 3. While it is anticipated that the model presented here will add to our understanding of the biological and inherent functional role of immune system TSHβv in human health, it should be noted that caution is warranted when ascribing splice site locations using predictive information (34, 80, 81). Even sequences that have a high potential as a splice site may be pseudosplice sites with limited functional significance. Sequence analyses may help to resolve this. Additionally, it may be possible to directly assess the functional role of TSHβv in a number of ways. In mice, for example, it may be possible using Crispr/Cas9 gene editing to selectively disrupt the expression of intron 4 and exon 5 in order to eliminate the TSHβv splice variant without affecting expression of a native TSHβ. An approach such as this, if successful, would permit experimental analyses into the extent to which immune system TSHβv contributes to thyroid biology and overall host metabolism. Although parallel studies cannot be done in humans, a version of that could be addressed *in vitro* using human cell lines. In summary, it is hoped that the system laid out here will provide a point from which to launch additional experiments into the role of TSHβv in health and disease.

## Author Contributions

The author confirms being the sole contributor of this work and has approved it for publication.

### Conflict of Interest Statement

The author declares that the research was conducted in the absence of any commercial or financial relationships that could be construed as a potential conflict of interest.
